# Safely disposing unused and unwanted prescription and over-the-counter medications: a public health, housing, and safety partnership in Framingham, MA

**DOI:** 10.1186/s40545-022-00407-1

**Published:** 2022-03-01

**Authors:** Tamara Vehige Calise, Sarah Levin Martin, Chloe Wingerter

**Affiliations:** 1 JSI Research and Training Institute, Inc., 44 Farnsworth Street, Boston, MA 02210 USA; 2 University of Maine at Farmington, Farmington, ME USA

**Keywords:** Drug take-back program, Unused drugs, Police-resident connections, Diverse communities, Older adults, Children/teens

## Abstract

**Background:**

The improper disposal of unused drugs can harm the environment and living beings. Programs such as drug take-back bins encourage people to dispose of unused medication at designated locations have increased. Unfortunately, awareness and participation is low, especially in ethnically and culturally diverse communities. The purpose of this paper is to describe the implementation of the Knock Talk and Toss (KTT), a drug take-back program aimed at taking unused drugs out of circulation and building police-resident connections in the housing authority of Framingham, MA.

**Methods:**

Multi-lingual brochures on the dangers of unused drugs and safe disposal were distributed on residents’ doors via the police department and/or listservs to residents living in the housing authority. Awareness efforts were then followed-up by teams of individuals, including the police, going door-to-door to collect any unused drugs, no questions asked. During the visits, one team member observed resident characteristics, whether drugs were disposed, including the estimated quantity and type, and police/resident interactions. Interviews were conducted with key staff and Chi-square analyses were used to assess socio-demographic differences in proportions of individuals willing to toss drug(s).

**Results:**

A total of 27 h were spent going door-to-door and 33 pounds of drugs were disposed. Households with observed adults aged 65 years or older and children/teenagers were twice as likely to dispose drugs compared to households, where these populations were not present.

**Conclusion:**

Initiatives, such as KTT, where police go door-to-door in areas with a higher concentration of families and elderly may help take unused drugs out of circulation while also enabling the police to have a positive presence in the community.

## Background

The use of both prescription (Rx) and over the-counter medications (OTC) has increased over the past few decades as a result of their availability; growing social acceptance; heightened awareness of their associations with solving problems; and the perception that they are safe, especially among young people [1, 2]. Moreover, the population is aging and living longer. Per Statista, the percentage of adults 65 and over represented 16.9% of the population in 2020, up from 11.3% in 1980. Research has found that almost 40% of older adults ages 65 and older take five or more Rx medications [3]. In a study of just over 3000 adults aged 57–85 years, researchers found that 81% used at least 1 Rx medication, 42% used at least 1 OTC, and 49% used a dietary supplement [4]. In this same study, 29% used at least 5 prescription medications concurrently. According to estimates listed on Statista, the total number of retail prescriptions filled annually in the United States increased from 4.07 billion in 2016 to 4.69 billion in 2021). Similarly, Consumer Healthcare Products Association reports that retail sales of OTC medicines have also increased, going from about $5.5 billion in 1980 to $36.5 billion in 2020.

The World Health Organization considers medication use to be sensible when taken appropriately, in doses that meet individual requirements, and for an adequate periods of time. However, irregular and inconsistent use of medication is common [5, 6], and for many reasons, medicines are prescribed, dispensed or sold inappropriately [7]. For these and other reasons, most consumers are left with some unused medicines at one time or another [8].

Unused medications in the home has increasingly caused concern due to its implications regarding accidental overdose; diversion, or selling of prescription drugs; and environmental safety. According to the American Association of Poison Control Centers (AAPCC), the overall rate of poison exposures reported to AAPCCs in 2019 was 643/100,00, and children aged 5 years or younger accounted for 42.8% [9]. Unintentional exposures were more common than intentional exposures in all age groups with the exception of ages 13–19 years of age; 27.4% of all exposures within this age group were intentional compared to 18.9% overall [9].

Of particular concern is the abuse/misuse use of prescription pain medications, or opioids, which was the second most commonly reported drug according to the National Institute on Drug Abuse. In a report prepared for the Substance Abuse and Mental Health Services Administration (SAMHSA) there were 9.9 million prescription pain reliever misusers 12 years of age and older in 2018, compared to, for example, 808,000 heroin users. This same report indicated that people aged 12 or older who misused painkillers reported getting them from a friend or relative—some got them for free (39%), some bought the drugs (10%), and others took them without asking (3%). Evidence suggests that many of the drugs that find their way into the general population are misappropriated from patients who received the original prescription for a legitimate medical purpose [10]. Another growing concern is misuse of pet medications [11, 12]. Although analgesics are thought to be the most common drug class diverted, studies suggest veterinarian opioid prescribing rate is increasing [11]. In a survey of 189 veterinarians, 44% were aware of opioid abuse or misuse by either a client or a veterinary practice staff member and 12% reported staff opioid abuse and diversion [12].

Finally, studies suggest that far too many consumers are unaware of proper disposal and wrongly throw medicines in garbage, toilet, or sink [13, 14]. Studies conducted in the U.S. found that less than 1% of people return unused medications to the pharmacy [15] and more than 50% flush them down the toilet [16] or in the trash [17]. International studies have found similar trends, where unwanted drugs are commonly thrown in the garbage [18–21]. Insani et al. [20] found that 82.1% of respondents threw unwanted drugs in the household garbage, 79.5% never received information about proper medication disposal practices, and 53.1% were unaware that unsafe medication disposal could harm the environment and population health. The improper disposal of unwanted and unused Rx and OTC medicines has been shown to contaminate the environment and pollute the nature, alter the food chain and harm the living beings [22, 23].

For these and other reasons, federal and state entities have sponsored or implemented collection programs, such as prescription drug take-back bins, events, media campaigns, and mail-back options to encourage people to anonymously dispose of unused or excess medication at designated locations, where drugs can be collected and incinerated [24–26].

While these efforts are thought to be suitable ways to remove unused Rx and OTCs from the home, awareness and participation in regulated collection activities is low, limiting the impact of the programs on a community-level [25, 27–29]. In a study of culturally and linguistically diverse suburban communities, Kearney et al., [27] found that less than one-third (30%) of survey respondents had heard of drug-take back events, and only 10% had participated. Non-English speakers in these same communities were less aware of take-back opportunities, and reported stigmatization, fear of law enforcement, and other perceived threats. These authors suggest the importance of creating educational materials in a variety of languages and for the police to participate in take-back events so as to not intimidate community members, such as undocumented immigrants who may avoid contact for fear of deportation.

### Purpose

The purpose of this study was to implement and evaluate a unique approach to the drug take-back strategy in the housing authority of an ethnically diverse city. We aim to illuminate a variety of socio-demographic factors correlated with prescription medication waste and disposal.

### Setting and intervention

The Knock Talk and Toss (KTT) program was implemented in Framingham, Massachusetts, located between Boston and Worcester. According to the U.S. Bureau of the Census, Framingham has a population of 73,123 with 19.2% of residents < 18 years of age and 15.4% 65 years or older. It is a linguistically and ethnically diverse community, with a higher percentage of residents who speak a language other than English in the home as compared to the state (39.5% vs. 23.6%, respectively). Framingham also has a higher percentage of Hispanic/Latino (16.1%) and Asian (8.0%) residents compared to Massachusetts (12.4% and 7.2%, respectively), and is known to be the home of the most recent Brazilian immigrants [30]. According to City-Data, Framingham is among the top cities with the most residents born in Brazil (#4 nationwide) with approximately 9% of the total population identifying as Brazilian. In addition to being a diverse community, Framingham is also ranked below average in terms of safety. The FBI Uniform Crime Reports shows the violent crime rate in Framingham has increased from 304 per 100,000 population in 2008 to 349 in 2018 (a 13.8% increase), whereas the rate in Massachusetts has decreased from 464 per 100,000 to 338 at these same points in time. Within Framingham, the Framingham Housing Authority (FHA) offers housing to low-income families, disabled persons and families, elderly, and elderly persons raising young children.

JSI Research & Training Institute (JSI) Healthy Communities, a public health non-profit, developed the KTT program in collaboration with the Framingham Police Department (FPD) and FHA. The goals of KTT were to: (1) increase awareness of the harmful effects of opioids, as well as proper ways to dispose of any unwanted, or unused, RX or OTC medication; (2) reduce any barriers related to proper disposal of medications (e.g., awareness, transportation to a drug take-back kiosk, stigmatization, etc.); and (3) build positive police and community relations within FHA.

**Fig. 1:**
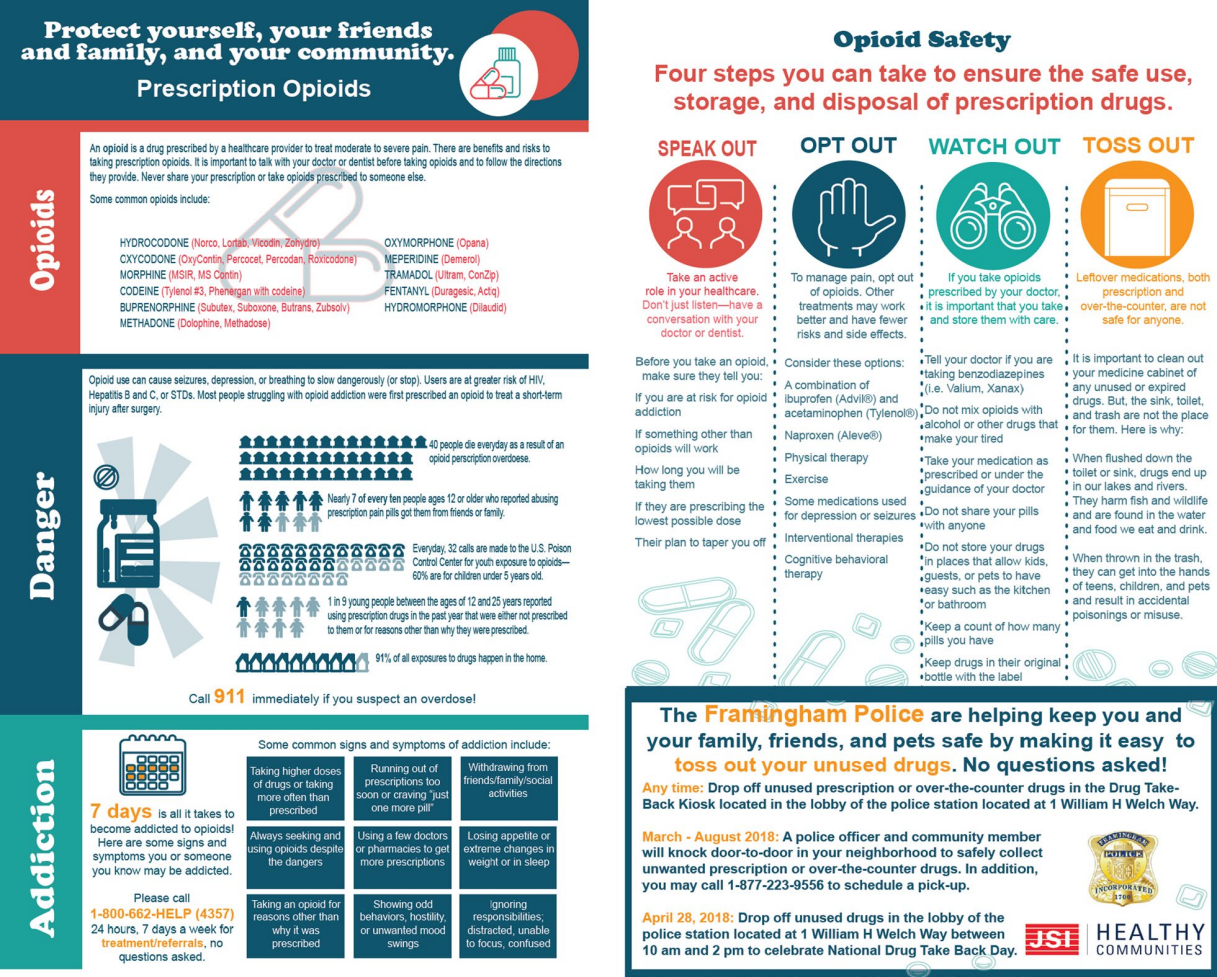
English Brochure front and back

JSI developed an 11 × 14, brightly colored brochure (Fig. 1) which included messages from evidence-based initiatives, such as the American Medicine Chest Challenges and National Drug Take-Back. The brochure was translated into Spanish, Portuguese, and Chinese (the three most commonly spoken languages in the FHA), and distributed in-person or via electronic listservs in all four languages to each household prior to the door-to-door knocks. In addition to important safety information, the brochures informed the residents that teams would be stopping by on a specified date to take any unwanted or unused RX or OTC medications with no questions asked.

During the door-to-door visits, a team made up of an FPD officer (the FHA police representative) and JSI evaluator went door-to-door in the areas, where the materials were previously distributed. An FHA bilingual staff member also accompanied the team in areas known to have more non-English speaking residents. When someone answered, the team showed the brochure to remind the resident of their visit and to see if they had any medications to dispose. Throughout the visits, both the FPD and FHA staff had an open dialog with residents to hear their concerns and answer questions they may have (related to KTT or more generally). Upon completion of the door-to-door knocking, the FPD and the evaluator double-sealed the drugs in two evidence bags for safety which they signed on the seal and weighed using a MEASURETEK 12R979 Digital Shipping Scale.

## Methods

The role of the JSI evaluator was to accompany the FPD (and FHA) and make observations based on a structured observation form which captured whether contact was made (e.g., no one home or person home). If someone was observed in the home, the observer marked if: (1) communication occurred; (2) language barriers prevented a conversation; (3) resident refused to talk/would not answer the door; or (4) the person home was not of legal age (≥ 18 years of age or older). When someone answered the door, the observer indicated the language spoken, the age of anyone observed (e.g., ≥ 65 years, child/teen), physical disability, and if resident chose to discard any drugs. If a resident discarded drugs, the observer indicated the type (Rx, OTC, or both) and how many drugs were discarded (< 10, 10–29, 30–49, 50–99, 100–199, or ≥ 200 pills). Finally, the observer indicated how much time was spent at the home and noted interactions between the FPD/FHA and the resident.

Data were entered in Survey Monkey and downloaded into Excel for analyses using JASP 0.9.2 accessed online at https://jasp-stats.org/. The primary outcome of interest was whether residents tossed unused drugs during the visit or not. Chi-square analyses was used to assess differences in proportions of individuals willing to toss drug(s). Differences in proportions were determined by non-overlapping 95% confidence intervals within significant Chi-square results. Descriptive analyses were conducted to describe respondent/household characteristics. Authors reviewed the qualitative information and summarized the themes, where relevant. Finally, the JSI evaluator conducted four interviews with FHA staff and the Chief of Police to better understand their motivations for implementing/supporting KTT.

## Results

The FPD and JSI team conducted door-to-door visits on 14 different dates between April and August, 2018 for a total of 27 h (average outing lasted approximately 75 min). The majority of the visits took place on different days throughout the week between 3 p.m. and 7 p.m.; two were conducted on Saturdays between 10 a.m. and 2 p.m. On eight of these days, one of three FHA staff joined the team, based on the demographic make-up of the targeted neighborhood. Two of the three FHA staff were Brazilian and spoke English, Spanish, and Portuguese, and one was Chinese and spoke English and Chinese. Overall, 33 pounds of Rx and OTC pills (ranging from 1.2 pounds to 9 pounds per outing) were disposed.

**Fig. 2:**
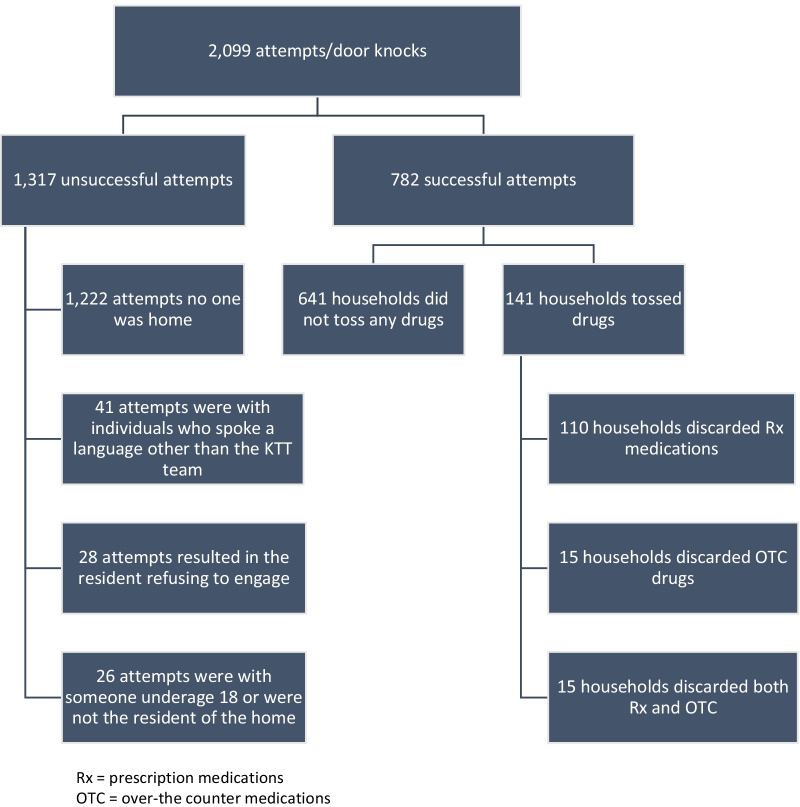
Attempts and results

Among the 2099 attempts to collect drugs, 782 successful contacts (37.2%) occurred; 1,222 (58.2%) attempts were unsuccessful, because no one was home; 41 attempts (2.0%) failed because language barriers prevented the conversation from happening; 28 attempts (1.3%) resulted in no contact, because the person home refused to engage (e.g., there was evidence that someone was home but they would not answer the door); and 26 attempts (1.2%) failed because the person home was underage and/or not the resident of the home (Fig. 2). Residents in majority of the households, where contact was made spoke English (83.9%); 7.7% spoke Spanish; 3.6% Chinese; 2.8% spoke Portuguese; and 1.9% spoke a different language and/or were deaf.

Overall, 641 of responding households (81.9%) did not toss any drugs, meaning they did not give the FPD drugs to dispose of correctly at the time of the visit. While the FPD and JSI team did not ask questions, respondents often provided reasons as to why they did not have any drugs to toss including that they took all of their medications and, therefore, did not have any to toss. Numerous people stated that they threw out their unused drugs (via the trash or in the toilet). Some said that they, or their caregiver, took unused drugs to the take-back kiosk in the hospital or police department, and others said to come back, they would have some to give at a later date.

Among the 141 households, where residents tossed drugs meaning they gave unused drugs to the FPD to dispose of correctly, 110 discarded Rx medications (14.1%), 15 discarded OTC drugs (1.9%) and 16 (2.1%) disposed both Rx and OTC drugs (Fig. 2). Just over one-third of the households (38.3%) tossed an estimated 30 to less than 100 pills and almost one-quarter (23.4%) discarded 100 or more pills. Very few of the households who disposed drugs (7.8%) tossed less than 10 pills. Of those who spoke a language other than English, significantly more disposed of drugs (20.8%; 95% CI: 14.6–28.8%) compared with those who spoke English (16.8%; 95% CI: 14.2–19.8%).

During the visit, the evaluator indicated if she observed an adult 65 years or older, child or teen in the residence, a notable disability, or a pet. Households with one or more of these characteristics were more likely to toss Rx or OT medication, compared to the 39 households were none of these were observed (11.1%; 95% CI: 8.2–14.9%). Among households where data collectors observed an adult aged 65 years or older, 62 (23.3%; 95% CI: 18.6–28.8%) tossed one or more drugs; where there was evidence of a child or teen, 20 (24.1%; 95% CI: 16.1–34.4%); where a disability was observed, 10 (20.0%; 95% CI: 11.1–33.2%) or a pet observed, 8 (14.8%; 95% CI: 7.4–26.9%).

Among all four FPD and FHA staff who were interviewed, there was a general consensus that the KTT offered opportunities for their staff to positively connect with residents. Regardless as to whether the respondent discarded drugs, residents were thankful that the FPD was implementing this initiative. Comments were observed in almost 10% of the households where contact was made such as “*this is great you guys are coming. Thank you! This is so awesome!*,*”* “*I appreciate what you are doing,” “This is brilliant,” “It’s a good thing you are doing,” “Thanks for coming around. I appreciate it,” “I am very grateful for you doing this, thank you,” and “I live in [city] and they don’t do this, this is a great program.”* While there were no negative interactions, there were a number of instances, where an especially positive interaction occurred between the FPD/FHA and the resident. For example, during numerous visits, the FPD connected with youth regarding sports and other activities. Several residents indicated that *“it was good to meet the FPD Housing Representative.”* At other times, FHA connected with residents and were able to see firsthand problems that needed to be addressed (e.g., broken lights, etc.).

## Discussion

Proper disposal of Rx and OTC drugs has been highlighted as an important step to reduce contamination of the environment and harm to living beings [13, 27]. We collected unused medications in the FHA to describe disposal within the housing authority of an ethnically diverse city, as well as the implementation of a take-back program by the police.

The KTT days and times were dependent upon the availability of the FPD, evaluator, and FHA staff. While the flyers were distributed door-to-door during the officer’s shift, the door-to-door knocking had to occur after his shift was over, and for safety reasons during daylight hours. The typical shift occurred between the hours of 3 pm and 7 pm, with several Saturday shifts, in an effort to catch more people while they were home. Unfortunately, we were only able to have contact with just over one-third of the residents as the majority were not home. Even with low contact rates, over a pound an hour was averaged during KTT (~ 1.33 pounds). Future door-to-door knocking should occur during different times a day (e.g., morning shifts).

Language is a consistent and significant barrier towards awareness of prevention options, as well as perceptions about community substance use. Kearney et al., [27], suggested the importance of using a multilingual approach, so that people who speak a language besides English may better understand the issue of prescription drug misuse and proper disposal practices. In our study, a higher proportion of residents speaking a language other than English tossed one or more drugs compared to English speaking residents, though given the wide confidence interval, the difference was not statistically significant. Our materials were in three other predominant languages and the multi-lingual FHA staff helped to explain the issues during our interactions. Unfortunately, there were times when the FHA staff were unavailable to do the door-to-door knocking and/or the staff who did join did not speak the same language (e.g., the FHA staff spoke Spanish but the resident spoke Chinese or vice versa). Future efforts should be made to further engage multi-lingual residents.

Older adults ages 65 and older are responsible for more than 34% of Rxs and 30% of OTC medicines taken (The State of Aging and Health in America, 2004), and more than 60,000 children are taken to emergency departments for evaluation from accidental poisonings every year [31]. We found that residents, where these two populations were observed, were significantly more likely to dispose Rx or OTC medications compared to households, where people over 65 years of age or a children/teens were not witnessed. Households with these two populations tossed at twice the rate of those households without these observed residents. The majority of the households (89.4%) disposed Rx medications which is not surprising given that 70% of the 200 most prescribed drugs are for conditions impacting the cardiovascular, central nervous, endocrine, and musculoskeletal systems [32], and age plays a vital role in the deterioration of cardiovascular functionality, physiological organ function, and other conditions which impact these systems [33, 34]. Stewart et al. [35], and Ma et al. [24] found that cardiovascular drugs accounted for the largest proportion of returns. Similarly, while we did not collect the type of Rx drug, residents often commented that they had heart problems, cancer, and diabetes, and changes to their prescriptions resulted in unused cardiovascular-related medications.

According to the 2018 Annual Report of the American Association of Poison Control Centers’ National Poison Data System (NPDS) [36], there has been a decline in the number of accidental poisonings among children which is thought to be a result of prevention efforts to educate parents. In the flyer we distributed, we provided multi-lingual messages about the dangers of opioids and Rx drugs to children, and ways to safely dispose of them. While we did not assess why more households with people 65 years or older or with children/youth, Kearney et al. [27], found the multi-lingual outreach and personal connections to be helpful in community engagement. Nevertheless, our findings suggest that efforts to further prevent harmful effects of improper disposal of Rx and OTC medications should target residents within these two age groups.

Finally, in addition to helping to safely dispose unwanted Rx and OTC medications, this intervention was implemented to build relationships among FPD and residents living in the FHA. Positive interactions were observed between residents and the FPD. In addition to having numerous informal conversations about the “comings and goings” in the community, residents connected with the FPD and many were appreciative of the program.

This initiative is not without limitations. From an implementation perspective, leaders within the community, such as the police department and housing authority, must see the value of such an initiative. This pilot would not have been possible without their support. That said, KTT was grant funded and in order for it to be sustained, dollars need to be allocated to staffing (e.g., paying over-time to police officers and staff) and supplies. We required an additional person to go door-to-door to ensure safe handling of the drugs, but this may not be required by others implementing such an initiative. The time within which the FPD FHA representative was available was limited to hours after 3 p.m., his typical shift, but before dark. In the future, there may be more contacts if the hours varied to include morning and early afternoon.

In terms of the evaluation, the age of residents was observed by the evaluator. It is possible that “younger looking” older adults may not have been classified as 65 years or older; similarly, others younger than 65 may have been categorized as older. While this is a limitation, the same evaluator conducted all of the observations. The grant funds were dedicated to the implementation of this initiative (not the evaluation), and as such, surveys and other efforts to assess residents’ perceptions, beliefs, and behaviors were not possible. Future efforts to understand the impact of KTT at the individual-level would be beneficial. Finally, this was a small, limited initiative and the generalizability to other communities should be taken with caution.

While there were limitations, this initiative took the drug-take back to another level by having the FPD go door-to-door in the housing authority. Given the observed comments, many of the residents that were engaged did not know how to safely dispose unwanted drugs and/or were not able/willing to go to the police station to do so. As such, the FPD helped to take unused drugs out of circulation, while they attempted to have a positive presence in the community. These findings suggest the importance of increasing awareness among the general population, especially more vulnerable households, on the importance of disposing unwanted drugs and ensuring everyone (e.g., including those who may have transportation limitations) has access to do safely do so. Cross-sector partnerships, such as public safety, housing, and health may all have an interest in these efforts.

## Conclusion

Initiatives, such as KTT, where police go door-to-door in areas with a higher concentration of families and elderly may help take unused drugs out of circulation while also enabling the police to have a positive presence in the community.

## Data Availability

The data sets generated during and/or analyzed during the current study are not publicly available given the small sample size but are available from the corresponding author on reasonable request.

## References

[1] McCarthy M (2007). Prescription drug abuse up sharply in the USA. Lancet.

[2] Holmstrom IK (2014). Swedish teenagers and over-the-counter analgesics—responsible, casual or careless use. Res Social Adm Pharm.

[3] Kantor ED (2015). Trends in prescription drug use among adults in the United States from 1999–2012. JAMA.

[4] Qato DM (2008). Use of prescription and over-the-counter medications and dietary supplements among older adults in the United States. JAMA.

[5] Schulz M (2016). Medication adherence and persistence according to different antihypertensive drug classes: a retrospective cohort study of 255,500 patients. Int J Cardiol.

[6] Krueger K (2018). In search of a standard when analyzing medication adherence in patients with heart failure using claims data: a systematic review. Heart Fail Rev.

[7] Manchikanti FB, Ailinani H, Pampati V (2010). Therapeutic use, abuse, and nonmedical use of opioids: a ten-year perspective. Pain Phys.

[8] Holloway KavDL. The World Medicines Situation 2011. Rational Use of Medicines. 2011, World Health Organization.: Geneva.

[9] Gummin DD (2020). 2019 Annual Report of the American Association of Poison Control Centers’ National Poison Data System (NPDS): 37th Annual Report. Clin Toxicol (Phila).

[10] Shrank WH (2011). Our bulging medicine cabinets–the other side of medication nonadherence. N Engl J Med.

[11] Anand A, Hosanagar A (2021). Drug misuse in the veterinary setting: an under-recognized avenue. Curr Psychiatry Rep.

[12] Mason DS (2018). Prescription opioid epidemic: do veterinarians have a dog in the fight?. Am J Public Health.

[13] Sonowal S, Desai C, Kapadia J (2016). A survey of knowledge, attitude, and practice of consumers at a tertiary care hospital regarding the disposal of unused medicines. J Basic Clin Pharmacy.

[14] Glassmeyer ST (2009). Disposal practices for unwanted residential medications in the United States. Environ Int.

[15] Bates C (2011). Overprescription of postoperative narcotics: a look at postoperative pain medication delivery, consumption and disposal in urological practice. J Urol.

[16] Seehusen DA, Edwards J (2006). Patient practices and beliefs concerning disposal of medications. J Am Board Fam Med.

[17] Law AV (2015). Taking stock of medication wastage: unused medications in US households. Res Social Adm Pharm.

[18] Bashaar M (2017). Disposal practices of unused and expired pharmaceuticals among general public in Kabul. BMC Public Health.

[19] Hassan MM (2008). Pattern of medical waste management: existing scenario in Dhaka City, Bangladesh. BMC Public Health.

[20] Insani WN (2020). Improper disposal practice of unused and expired pharmaceutical products in Indonesian households. Heliyon.

[21] Sasu S, Kummerer K, Kranert M (2012). Assessment of pharmaceutical waste management at selected hospitals and homes in Ghana. Waste Manag Res.

[22] Daughton CG, Pharmaceuticals in the environment: sources and their management. Analysis, fate and removal of pharmaceuticals in the water cycle., ed. Petrovic M and B. D. 2007: Elsevier Science. 1–58.

[23] Ruhoy IS, Daughton CG (2007). Types and quantities of leftover drugs entering the environment via disposal to sewage–revealed by coroner records. Sci Total Environ.

[24] Ma CS (2014). Drug take back in Hawai’i: partnership between the University of Hawai’i Hilo College of Pharmacy and the Narcotics Enforcement Division. Hawaii J Med Public Health.

[25] Adler AC (2020). Mail-back envelopes for retrieval of opioids after pediatric surgery. Pediatrics.

[26] Yanovitzky I (2016). The American medicine chest challenge: evaluation of a drug take-back and disposal campaign. J Stud Alcohol Drugs.

[27] Kearney M (2019). Primary prevention of prescription drug misuse among culturally and linguistically diverse suburban communities. J Community Health.

[28] Lewis ET, Cucciare MA, Trafton JA (2014). What do patients do with unused opioid medications?. Clin J Pain.

[29] Lystlund S (2014). Patient participation in a clinic-based community pharmacy medication take-back program. J Am Pharm Assoc (2003).

[30] Lima A, S.C., Brazilians in the U.S. and Massachusetts: a demographic and economic profile, Paper 50. 2007: Gaston Institute Publications.

[31] Ferguson R. Keeping families safe around medicine. 2014: Washington: Safe Kids Worldwide; 2014.

[32] Fuentes AV, Pineda MD, Venkata KCN (2018). Comprehension of top 200 prescribed drugs in the US as a resource for pharmacy teaching, training and practice. Pharmacy (Basel).

[33] Aunan JR, Cho WC, Søreide K (2017). The biology of aging and cancer: a brief overview of shared and divergent molecular hallmarks. Aging Dis.

[34] Rodgers JL (2019). Cardiovascular risks associated with gender and aging. J Cardiovasc Dev Dis.

[35] Stewart H (2015). Inside Maine’s medicine cabinet: findings from the drug enforcement Administration’s medication take-back events. Am J Public Health.

[36] Gummin DD (2019). 2018 Annual Report of the American Association of Poison Control Centers’ National Poison Data System (NPDS): 36th Annual Report. Clin Toxicol (Phila).

